# The Journal of Laryngology and Rhinology: An Analytical Record of Current Literature Relating to the Throat and Nose

**Published:** 1887-03

**Authors:** 


*The Journal of Laryngology and Rhinology: an Analytical Record of Current Literature relating to the Throat and Nose.* Edited by MORELL MACKENZIE and R. NORRIS WOLFENDEN, assisted by able Coadjutors in this and other Countries. London: J. & A. CHURCHILL. No. 1. January, 1887.

We are pleased to welcome a new English journal, which deals with the important literature relating to the throat and nose. It is conducted on the plan of a German *Centralblatt*, and aims at analysing every article dealing with Laryngology and Rhinology, no matter in what language it may have been originally written. It is evident that it has a *raison d'être* in the fact that since the *Archives of Laryngology* became extinct, there has been no journal covering the same ground and printed in English; and although the German paper edited by Semon, and the *Revue mensuelle de Laryngologie*, edited by Moure, are very valuable, they are not read by many in this country, because they are not written in our mother tongue. It is published monthly.

